# Severe Epistaxis Secondary to Dabrafenib and Trametinib Toxicity in Non-small Cell Lung Carcinoma With Small Bowel Metastasis

**DOI:** 10.7759/cureus.16431

**Published:** 2021-07-16

**Authors:** Keerthi Gullapalli, Osama Mosalem, Merryl T Varghese, Kevin Watat, Borys Hrinczenko

**Affiliations:** 1 Internal Medicine, Michigan State University - Sparrow Hospital, Lansing, USA; 2 Internal Medicine, Michigan State University College of Human Medicine, East Lansing, USA; 3 Hematology and Oncology, Michigan State University - Breslin Cancer Center, Lansing, USA

**Keywords:** epistaxis, dabrafenib, trametinib, lung carcinoma, braf v600e, hemorrhage, metastasis, non-small cell lung carcinoma (nsclc)

## Abstract

BRAF mutations are estimated to be present in 2-4% of non-small cell lung carcinoma (NSCLC) cases. BRAF inhibitor (dabrafenib) and MEK inhibitor (trametinib) are currently approved to treat NSCLC harboring the BRAF V600E mutation. However, the use of this new combined targeted therapy can be associated with severe and life-threatening toxicities.

Here, we describe the case of a 77-year-old male with a history of BRAF-positive lung adenocarcinoma with metastasis to the brain, adrenals, and small bowel (jejunum), currently on dual therapy with dabrafenib and trametinib, who presented with refractory epistaxis. The dual therapy regimen was started one month prior to his presentation. After initial stabilization with anterior nasal packing, intravenous and nebulized tranexamic acid (TXA) in the emergency department (ED), he suddenly developed respiratory decompensation. He needed emergent intubation for acute hypoxic respiratory failure and airway protection secondary to profuse bleeding. He was extubated 24 hours later as the epistaxis was manageable, and the nasal packing was removed. Shortly after extubating, he started coughing copious amounts of blood and developed respiratory distress with stridor requiring re-intubation. A large blood clot was noted to be partially occluding the vocal cords on laryngoscopy and was removed during intubation. An emergent flexible fiberoptic bronchoscopy was performed with the retrieval of a large blood clot extending from the oropharynx down into the distal trachea. There was no evidence of acute bleeding within the lung after the clot was removed. Workup to explore the cause of his bleeding included a coagulation profile, which was unrevealing. His bleeding was most likely consistent with a side effect of his treatment with dabrafenib and trametinib.

Life-threatening bleeding has been reported as a side effect of the combination therapy with dabrafenib and trametinib in metastatic melanoma. Also, in the phase 2 clinical trial (BRF113928) of dabrafenib plus trametinib in patients with previously untreated BRAF V600E-mutant metastatic NSCLC, 3.2% of subjects developed a grade III or IV hemorrhage. Our case aims to raise physicians' awareness of one of the significant side effects of this combination therapy especially since this combination is being used more frequently and now also in lung cancer.

## Introduction

In the past decade, lung cancer has not only occupied a prominent place among the most common cancers, but it is also the leading cause of cancer death [1]. In the United States (US), lung cancer accounts for about 14% of new cancers in men and 13% of new cancers in women [1]. This significantly high incidence has prompted further studies of the disease leading to its classification into two main subcategories: small cell lung cancer (SCLC) and non-small cell lung cancer (NSCLC). NSCLC accounts for 80 to 85% of new lung cancer cases [2]. NSCLC is further subdivided into large cell carcinoma (LCC), squamous cell carcinoma (SCC), and adenocarcinoma. Understanding the genetic distinction between these subcategories has proven invaluable in developing a more targeted therapeutic approach. In recent studies, NSCLC was shown to be linked to several genetic mutations such as mutations in epidermal growth factor receptor (EGFR), echinoderm microtubule-associated protein-like 4 (EML4), anaplastic lymphoma kinase (ALK) fusion oncogene, c-ROS oncogene 1 (ROS1) fusions and B-Raf proto-oncogene (BRAF) [3]. The latter is the target of many cancer therapies, of which the recent Food and Drug Administration (FDA)-approved dabrafenib-trametinib combination will be discussed in this report. Dabrafenib and trametinib target BRAF and MEK1/2, respectively. These genes encode proteins of the serine/threonine kinase family that are members of the intracellular signaling cascade known as the RAS/RAF/MEK/ERK pathway [4]. This pathway regulates cell proliferation, differentiation, migration, and apoptosis, making it a suitable target for cancer therapy. In the most recent FDA review, dabrafenib-trametinib combination therapy was shown to improve both the progression-free survival and overall survival of patients with NSCLC harboring BRAF V600E or V600K mutations [4]. However, many adverse reactions were also documented, leading to multiple treatment interruptions or dose reductions, of which pyrexia, fatigue, and nausea were reported as some of the most frequent adverse reactions [4]. Hemorrhage was also described as one of the reported side effects, however, the spectrum and severity of bleeding have been infrequently reported in the literature.

## Case presentation

A 77-year-old Caucasian male with a past medical history significant for hypertension and stage IV (American Joint Committee (AJCC) clinical staging) (cTx,cN0,pM1) left lung adenocarcinoma with malignant pleural effusion, metastasis to the brain, bone, adrenals, and small bowel status-post jejunal resection presented to the emergency department (ED) with intractable epistaxis. Of note, our patient's initial diagnosis of lung adenocarcinoma was from a metastatic small bowel mass (jejunal intraluminal mass), which is a very rare presentation. Comprehensive genomic profiling at diagnosis showed stable microsatellite instability (MSI), low tumor mutation burden (TMB), and presence of BRAF V600E mutation, without other mutations identified. He completed stereotactic brain irradiation for his brain metastasis and was started on the FDA-approved combination of dabrafenib/trametinib dual-therapy (DTT) one month before his presentation to us. This combination is targeted immunotherapy for BRAF positive and advanced metastatic disease. He reported a four-day history of intermittent, self-limiting epistaxis, which became uncontrollable on the day of his presentation. In the ED, he was profusely bleeding from bilateral nares and endorsed difficulty in breathing. He was visibly distressed, and the initial vital signs were significant for sinus tachycardia with a rate of 157 beats/min, blood pressure 143/107 mm of Hg, respiratory rate of 22 per minute, and oxygen saturation of 100% on room air. He received nebulized and intravenous tranexamic acid (TXA) along with anterior nasal packing and suctioning of the bloody secretions. Later, while in the ED, he became hypoxic with his oxygen saturation dropping to 72% on room air. He was in respiratory distress and could not protect his airway; thus, he needed emergent intubation. Relevant lab values at admission are displayed in Table 1. 

**Table 1:** Table showing the patient's initial labs on presentation to the hospital

S. No.	Labs on admission (units)	Value	Reference range
1.	Hemoglobin (g/dl)	8.7	12.6-16.5
2.	Platelet (cells x 10^3^/mm^3^)	246	150-400
3.	Prothrombin time (seconds)	9	9-11.5
4.	International normalized ratio (INR)	0.9	
5.	Activated partial thromboplastin time (aPTT) (seconds)	20.1	21-31

**Figure 1:**
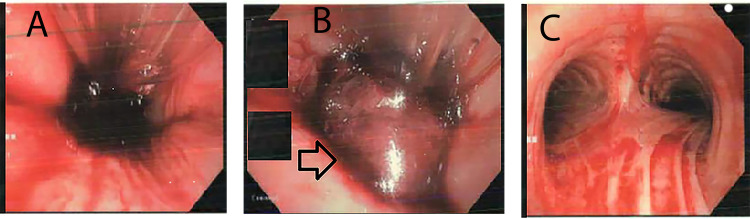
Flexible fiberoptic bronchoscopy images. (A) Diffuse bleeding from the airway. (B) A large blood clot (Arrowhead) obstructing the trachea. (C) Diffuse airway erythema without active bleeding following clot removal

**Figure 2:**
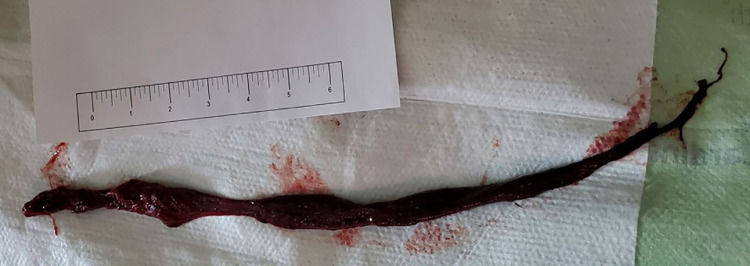
Large blood clot removed by bronchoscopy measuring approximately 12 inches

His medication list was thoroughly reviewed, and he was not found to be on any antiplatelet or anticoagulation medications. His epistaxis was controlled with an anterior nasal pack (which was eventually removed the next day) and he was extubated 24 hours later. However, immediately after extubation, he started coughing copious amounts of dark clotted blood and bright red fresh blood along with the development of stridor. His oxygen saturation dropped to 59% on room air and he was noted to be in acute hypoxic respiratory distress requiring emergent re-intubation. During re-intubation, laryngoscopy showed a large blood clot around the vocal cord folds. Bronchoscopy was done emergently following intubation which revealed a large blood clot extending from the oropharynx down into the distal trachea with no evidence of active bleeding following clot removal (Figure 1). The clot measured approximately 12 inches (Figure 2). 

His coagulation profile was essentially normal, thus ruling out other causes of bleeding. The hematology-oncology hospital team was consulted for an expert opinion; they acknowledged that bleeding was likely cancer treatment-related and recommended treatment interruption. He was eventually extubated and did not have any further bleeding episodes. After discussion with the patient, family, and treatment teams, he was transferred to hospice care and thus discharged to a hospice facility.

## Discussion

The management of NSCLC has dramatically changed over the last few years with the discovery of new targeted therapies. Mutations in the proto-oncogene BRAF are rare in lung cancers and are considered present in only 2-4% of NSCLC cases, while MEK mutations can be present in only 1%. The majority of BRAF mutations were found in lung adenocarcinoma (97%) versus squamous cell carcinoma (3%) [5,6]. The most commonly described BRAF mutation responsible in NSCLC is the BRAF V600E point mutation, which accounts for almost 50% of the BRAF mutations in NSCLC cases [7,8]. Mutations in BRAF oncogene lead to persistent activation of cell signaling, uncontrolled cell growth, and proliferation [8]. The discovery of this point mutation led to the development of novel selective inhibitors against BRAF mutations. However, cancer cells can evade this through bypass pathways such as MEK1/MEK2 kinases [9]. The addition of a MEK inhibitor to BRAF inhibition has improved the clinical outcomes in some cases [9]. Molecular testing for BRAF mutations is now part of the primary workup for lung cancer according to the National Comprehensive Cancer Network (NCCN) guidelines. In 2017, dabrafenib (a BRAF inhibitor) and trametinib (a MEK Inhibitor) were approved by the FDA for use in newly diagnosed or previously treated patients with BRAF mutations [4,10]. Odogwu and colleagues reported pyrexia, fatigue, and nausea as some of the most frequent adverse reactions, which occurred in almost 50% of the treatment cohort in their clinical trial [4]. Most of these adverse reactions were managed by modifications to dosing levels and temporary interruptions. In the phase 2 clinical trial (BRF113928) of dabrafenib plus trametinib in patients with previously untreated BRAF V600E-mutant metastatic NSCLC, 3.2% of subjects developed a grade III or IV hemorrhage [10]. Bleeding was described as hemoptysis, gastrointestinal hemorrhage, injection site hematoma, or epistaxis [4,10]. According to a literature search on Medline, two case reports have described severe bleeding secondary to the combination treatment with dabrafenib and trametinib [11,12]. The etiology of bleeding was unclear; however, it was hypothesized to be related to rapid tumor response to treatment [11]. It is very well known that platelets express several members of the mitogen-activated protein kinase (MAPK) signaling cascade, including MEK1 and MEK2, and their downstream effectors ERK1 and ERK2 [13,14]. Some studies have reported the role of the MAPK signaling pathway in platelet aggregation, adhesion, and thrombus formation [14,15]. Some in vivo studies on mice have also demonstrated inhibition of thrombus formation and thrombus destabilization following injection with a MEK inhibitor [14,16]. It was thus hypothesized that inhibition of MAPK signaling by dabrafenib and trametinib could be the reason for bleeding side effects in these patients. However, Unsworth and colleagues revealed in their study that MEK inhibitors did not induce platelet dysfunction [14]. Despite this, there still exists much ambiguity on how significant of a role MAPK and subsequently MEK inhibition contribute to the causation of bleeding that is seen in these patients; it is an area for further research.

In our case, a normal platelet count and a normal coagulation profile ruled out bleeding diathesis. After the removal of his large clot, there was no evidence of tumor invasion into the bronchi nor an active source of bleeding. Therefore, we excluded other etiologies of bleeding in this case, and the combination of dabrafenib and trametinib remained the most likely etiology for his bleeding. The presence of a clot in his lung could be explained by nebulized TXA, which was used to control his bleeding at the time of admission.

Interestingly, our patient had a small bowel metastasis in association with a BRAF mutation. Small bowel metastasis is very uncommon in lung cancer in general and NSCLC in particular [17]. Qasrawi et al. described a case of BRAF mutant NSCLC associated with duodenal metastasis [17]. Marchetti et al. showed that V600E mutation represents a negative prognostic factor in patients with NSCLC. It is associated with aggressive histology, significantly shorter disease-free, and overall survival than those without mutations [6]. The previously reported cases with small bowel metastasis, as well as our case, suggest the aggressive behavior of BRAF mutant NSCLC with small bowel metastasis. To the best of our knowledge, this is the third reported case of well-documented BRAF-mutant lung adenocarcinoma with metastases to the gastrointestinal tract.

## Conclusions

Dabrafenib and trametinib are novel targeted therapies for BRAF V600E mutant NSCLC. Severe bleeding is a relatively uncommon side effect, and its management is challenging. BRAF mutant NSCLC is associated with aggressive behavior and poor prognosis. Our case aims to raise physicians' awareness about BRAF mutant NSCLC and the potential side effect of severe bleeding that is seen with this combination treatment regimen.

## References

[1] de Groot PM, Wu CC, Carter BW, Munden RF (2018). The epidemiology of lung cancer. Transl Lung Cancer Res.

[2] (2021). What Is Lung Cancer?. https://www.cancer.org/cancer/lung-cancer/about/what-is.html.

[3] Sholl LM (2015). Biomarkers in lung adenocarcinoma: a decade of progress. Arch Pathol Lab Med.

[4] Odogwu L, Mathieu L, Blumenthal G (2018). FDA approval summary: dabrafenib and trametinib for the treatment of metastatic non-small cell lung cancers harboring BRAF V600E mutations. Oncologist.

[5] Baik CS, Myall NJ, Wakelee HA (2017). Targeting BRAF-mutant non-small cell lung cancer: from molecular profiling to rationally designed therapy. Oncologist.

[6] Marchetti A, Felicioni L, Malatesta S (2011). Clinical features and outcome of patients with non-small-cell lung cancer harboring BRAF mutations. J Clin Oncol.

[7] Okimoto RA, Lin L, Olivas V (2016). Preclinical efficacy of a RAF inhibitor that evades paradoxical MAPK pathway activation in protein kinase BRAF-mutant lung cancer. Proc Natl Acad Sci U S A.

[8] Tissot C, Couraud S, Tanguy R, Bringuier PP, Girard N, Souquet PJ (2016). Clinical characteristics and outcome of patients with lung cancer harboring BRAF mutations. Lung Cancer.

[9] O'Leary CG, Andelkovic V, Ladwa R (2019). Targeting BRAF mutations in non-small cell lung cancer. Transl Lung Cancer Res.

[10] Planchard D, Smit EF, Groen HM (2017). Dabrafenib plus trametinib in patients with previously untreated BRAFV600E-mutant metastatic non-small-cell lung cancer: an open-label, phase 2 trial. Lancet Oncol.

[11] Flaherty DC, Hoffner BW, Lau BJ, Hamid O, Faries MB (2015). Hepatic hemorrhage as a consequence of rapid response to combined targeted therapy in metastatic melanoma. J Surg Oncol.

[12] Lee le M, Feun L, Tan Y (2014). A case of intracranial hemorrhage caused by combined dabrafenib and trametinib therapy for metastatic melanoma. Am J Case Rep.

[13] McNicol A, Shibou TS, Pampolina C, Israels SJ (2001). Incorporation of MAP kinases into the platelet cytoskeleton. Thromb Res.

[14] Unsworth AJ, Bye AP, Kriek N, Sage T, Osborne AA, Donaghy D, Gibbins JM (2019). Cobimetinib and trametinib inhibit platelet MEK but do not cause platelet dysfunction. Platelets.

[15] Garcia A, Quinton TM, Dorsam RT, Kunapuli SP (2005). Src family kinase-mediated and Erk-mediated thromboxane A2 generation are essential for VWF/GPIb-induced fibrinogen receptor activation in human platelets. Blood.

[16] Hiratsuka T, Sano T, Kato H (2017). Live imaging of extracellular signal-regulated kinase and protein kinase A activities during thrombus formation in mice expressing biosensors based on Förster resonance energy transfer. J Thromb Haemost.

[17] Qasrawi A, Tolentino A, Abu Ghanimeh M, Abughanimeh O, Albadarin S (2017). BRAF V600Q-mutated lung adenocarcinoma with duodenal metastasis and extreme leukocytosis. World J Clin Oncol.

